# Aneurysmal Wall Enhancement of Non-Ruptured Intracranial Aneurysms after Endovascular Treatment Correlates with Higher Aneurysm Reperfusion Rates, but Only in Large Aneurysms

**DOI:** 10.3390/diagnostics14141533

**Published:** 2024-07-16

**Authors:** Valentin Ladenhauf, Malik Galijasevic, Milovan Regodic, Raimund Helbok, Verena Rass, Christian Freyschlag, Ondra Petr, Johannes Deeg, Leonhard Gruber, Stephanie Mangesius, Elke Ruth Gizewski, Astrid Ellen Grams

**Affiliations:** 1 Department of Radiology, Medical University of Innsbruck, 6020 Innsbruck, Austria; valentin.ladenhauf@i-med.ac.at (V.L.); johannes.deeg@i-med.ac.at (J.D.); leonhard.gruber@i-med.ac.at (L.G.); stephanie.mangesius@i-med.ac.at (S.M.); elke.gizewski@i-med.ac.at (E.R.G.); astrid.grams@i-med.ac.at (A.E.G.); 2 Neuroimaging Research Core Facility, Medical University of Innsbruck, Anichstraße 35, 6020 Innsbruck, Austria; 3 Department of Neurology, Kepler University Hospital, Johannes Kepler University Linz, 4020 Linz, Austria; raimund.helbok@i-med.ac.at; 4 Department of Neurology, Medical University of Innsbruck, 6020 Innsbruck, Austria; verena.rass@i-med.ac.at; 5 Department of Neurosurgery, Medical University of Innsbruck, 6020 Innsbruck, Austria; christian.freyschlag@i-med.ac.at (C.F.); ondra.petr@i-med.ac.at (O.P.)

**Keywords:** neuroradiology, aneurysm, MRI, reperfusion

## Abstract

Introduction: Aneurysmal wall enhancement (AWE) of non-ruptured sacular intracranial aneurysms (IA) after endovascular treatment (ET) is a frequently observed imaging finding using AWE-sequences in brain magnetic resonance imaging (MRI). So far, its value remains unclear. We aimed to investigate the effect of AWE on aneurysm reperfusion rates in a longitudinal cohort. Methods: This is a retrospective MRI study over the timespan of up to 5 years, assessing the correlation of increased AWE of non-ruptured IAs and events of aneurysm reperfusion and retreatment, PHASES Score and grade of AWE. T1 SPACE fat saturation (FS) and T1 SE FS blood suppression sequences after contrast administration were used for visual interpretation of increased AWE. The IAs’ sizes were assessed via the biggest diameter. The grade of enhancement was defined in a grading system from grade 1 to grade 3. Results: 127 consecutive non ruptured IA-patients (58.9 ± 9.0 years, 94 female, 33 male) who underwent elective aneurysm occlusion were included. AWE was observed in 40.2% of patients (51/127) after ET, 6 patients already showed AWE before treatment. In large IAs (which were defined as a single maximum diameter of over 7.5 mm), AWE was significantly associated with aneurysm reperfusion in contrast to large aneurysm without AWE). All grades of AWE were significantly associated with reperfusion. Conclusions: Our data suggests that in patients with initially large IAs, AWE is correlated with aneurysm reperfusion.

## 1. Introduction

Intracranial aneurysms (IA) have a prevalence of 3–5%, with a yearly rate of rupture of around 1%. Aneurysm rupture is a life-threatening disease with high morbidity and mortality. In patients with known non-ruptured IAs—oftentimes incidentally identified during brain examinations for other reasons—precise evaluation of risk factors for rupture and reperfusion after occlusion via ET is crucial for decision making. Close follow up without aneurysm occlusion may be weighted against endovascular or surgical aneurysm occlusion, since aneurysm occlusion is known to be associated with a 2–5% complication rate [1,2].

Scoring systems like the “PHASES–SCORE” aim to quantify risk of rupture of IAs based on aneurysm morphology and basic patient information, one of the most important deciding parameters being size [3,4]. Although size of non-ruptured IAs is a primary deciding factor concerning therapeutic options, recent data showed that also small IAs can rupture [5]. This leads to the consensus that additional data concerning the stability or instability of IAs is needed.

High–resolution vessel wall imaging (HR–VWI) is an innovative MR imaging tool to visualize the wall of IAs. Multiple studies suggest a correlation between enhancement of vessel walls and inflammation of vessel walls, progression of the size of IAs and a potentially high risk of rupture in incidental IAs [6,7]. Chyatte et al. could show that aneurysm formation and rupture is associated with an inflammatory reaction of the vessel wall [8]. Histological work up of ruptured aneurysm tissue also showed an elevated rate of inflammatory cells, compared to non-ruptured aneurysm tissue [9,10]. Inflammatory vasculopathies and atherosclerosis show increased vessel wall enhancement [11,12]. HR–VWI can be helpful in identifying IA with increased risk of rupture [13,14,15].

Aneurysm reperfusion after endovascular treatment remains a challenging multidisciplinary situation in which radiological exams play a central role [16], with one often encountered subject being arterial wall enhancement in contrast enhanced MRI. Although part of recent literature [17,18] suggests that AWE of an IA following ET is a common finding and is most possibly associated with the healing process, other recent studies show different results [19,20], so the prognostic value of this finding remains unclear.

The aim of this study is to investigate if aneurysmal vessel wall enhancement of treated IAs correlates with higher aneurysm reperfusion rates. We hypothesized that AWE is associated with an increased risk of aneurysm reperfusion.

## 2. Methods and Materials

### 2.1. Research Design

This retrospective imaging study has been approved by the local ethics committee of the Medical University of Innsbruck, Austria (ECS 1089/2021) and was conducted in compliance with the Declaration of Helsinki.

### 2.2. Patients

Inclusion criteria were age above 18 years, detection of a non-ruptured IA, at least two full MRI studies with designated sequences designed for evaluation of aneurysmal wall enhancement (if available prior to any endovascular intervention and at least one further study after ET), history of at least one diagnostic subtraction angiography study which included endovascular aneurysm occlusion via coiling and clinical history including information of earlier subarachnoid hemorrhage (SAH) from another aneurysm, history of hypertension and ethnicity in order to calculate the individual PHASES Score. Exclusion criteria were prior history of SAH from a ruptured aneurysm, endovascular treatment via stent or stent—assisted coiling and known history of vasculitis or intracranial vascular malformation. The inclusion and exclusion criteria are visually stated in the following Table 1.

Patient data were extracted from our Picture Archiving and Communication System (PACS) and locally recorded clinical histories, HR–VWI sequences were assessed in the timeframe between 1 January 2015 and 31 December 2020.

### 2.3. Image Acquisition and Analysis

MR images of the brain for each patient were acquired using a 1.5 T (Siemens Aera, Siemens Healthineers, Erlangen, Germany) or a 3T MR imaging scanner (Siemens Skyra, Siemens Healthineers, Erlangen, Germany) with a standard 64-element head coil. Similar acquisition protocols were used for all MRI scans. The protocol included DWI and ADC–maps (b-values were 0 and 1000 s/mm^2^) in an axial plane, T2 turbo spin echo in an axial plane, contrast MR angiography with reconstructions, T1 time of flight angiography. For evaluation of aneurysmal wall enhancement, we used two sequences at our institution in the evaluated timespan. From 2015 to 2018 we used T1 Spin Echo Blood-suppression with fat saturation (T1 SE FS blood-suppression) before and after contrast administration, from 2018 to December 2020 we used T1 sampling perfection with application-optimized contrast using different flip angle evolutions and fat saturation (T1 SPACE FS) before and after contrast administration in sagittal orientation with reconstructions in axial and coronal planes. Gadolinium contrast agent was administered intravenously in a typical dose of 0.1 mL per kg of body weight, 4 min prior to acquisition of the contrast enhanced T1 SPACE FS and T1 SE FS blood-suppression sequences. Image quality of our MRI studies was standard, both sequences allow evaluation of AWE, mainly because of excellent spatial resolution and fat saturation.

The specific sequence parameters are summarized in Table 2.

### 2.4. Assessment of Intracranial Aneurysms in Imaging Sequences and Clinical History

We assessed the following features and measurements:

1. Presence and duration of aneurysmal wall enhancement of the electively treated aneurysms on MRI (if available prior to any intervention and every recorded scan after elective intervention including sequences for HR–VWI in the timespan of 1 January 2015 to 31 December 2020).
2. Analysis of aneurysm location on MRI and DSA.
3. Grade of AWE
4. Aneurysm recurrence/reperfusion and retreatment
5. Size of the aneurysm (maximum diameter in mm)
6. PHASES–Score, which includes
  (a) Age
  (b) Population (Finnish, Japanese, North American and European)
  (c) History of hypertension
  (d) Size of aneurysm
  (e) Site of aneurysm
  (f) Earlier subarachnoid hemorrhage from another aneurysm

One experienced neuroradiologist performed the assessment of IAs in MRI and DSA-studies in a first step, blinded to the written radiological reports, which were consulted in a second step. In case of disagreement, expert opinion was considered. Size parameters were strictly assessed retrospectively on DSA-studies. For the assessment of aneurysm recurrence/reperfusion we applied the Raimond Roy classification [21] which is based on the evaluation of residual inflow of intracranial aneurysms in DSA-studies. Although this classification is not routinely used for non-invasive depiction of intracranial vessels via MRI, aneurysm recurrence/reperfusion can likewise be distinguished between contrast filling of the aneurysm neck vs. aneurysm sac filling, which has implications concerning further treatment options [22].

### 2.5. Grading of AWE

**Figure 1:**
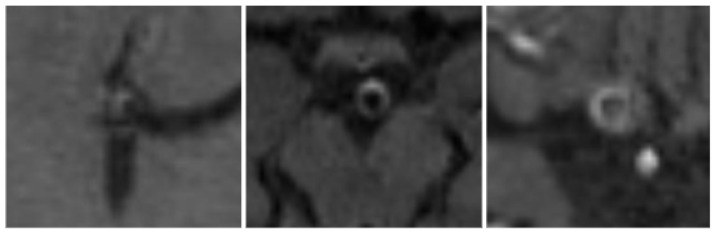
From left to right: **Left**: Grade 1 AWE in a middle cerebral artery Aneurysm, showing non-continuous arterial wall semilunar enhancement on the right ventral circumference. **Middle**: Grade 2 AWE in a basilar artery aneurysm showing continuous arterial wall enhancement without thickening of the vessel wall. **Right**: Grade 3 AWE in an internal cerebral artery Aneurysm with continuous enhancement and thickening of the vessel wall, the dorsolaterally located enhancing structure in the bottom right corner of the figure corresponds to the pituitary infundibulum.

In this study, if present, AWE was categorized into 3 grades, based on the enhancement patterns and enhancement intensity, combined with thickening of the vessel wall of IAs in the follow up examinations after ET, as shown in Figure 1:

- Grade 1: Non–continuous enhancement
- Grade 2: Continuous linear enhancement without thickening of the aneurysmal wall
- Grade 3: Continuous linear enhancement with thickening of the aneurysmal wall

### 2.6. Statistical Analysis

Statistical analysis was performed using R (R Core Team v. 3.3.0).

For demographic data, descriptive statistics were applied. Categorical data as count and percentage.

The differences of demographics between the two groups were compared using a non-parametric Mann-Whitney U test (MWU). Summary statistics were calculated including median with first and third quartiles (Q1–Q3). Contingency tables were analyzed using Fisher exact test. Statistical significance was defined as *p* < 0.01. Logistic regression was used to model the relationship between reperfusion binary outcome variable and a predictor variable, the *p*-values were reported. Spearman rank-order correlation between the variables was also checked.

A diagnostic performance was analyzed by a receiver operating characteristics (ROC) curve. The cut-off values were determined using the greatest Youden’s J index in order to calculate sensitivity and specificity.

Correlation between variables was also checked.

## 3. Results

127 patients were included in this retrospective study (median age 58.9 years (47.7 to 65.7)); 94 female (74.0%), 33 male (26.0%). 51 (40.2%) patients showed increased AWE after ET (median age 61.2 years (51.9 to 66.7); 38 female (74.5%), 13 male (25.5%)), 76 (59.8%) patients did not show increased AWE after ET (median age 55.4 years (45.9 to 64.3); 56 female (73.7%), 20 male (26.3%)).

Table 3 and Table 4 show the population demographics and comparison of PHASES scores and radiological features in patients with reperfusion and AWE (maximum aneurysm diameter is depicted in millimeters).

Seven (13.7%) patients had already shown AWE prior to any intervention in the baseline MRI with AWE persisting in all of them. Six of those seven patients showed reperfusion after endovascular treatment, two patients needed a second endovascular treatment. Unfortunately, the sample size of this group was too small to perform any statistical analysis.

44 of 51 patients (86.3%) firstly showed AWE after ET, with a median timespan of 18 months [1. quartil = 0.06 months (equal to 2 days)/3. quartil = 67.7 months] after ET.

In the group with AWE after ET, 37 of 51 (72.5%) patients showed reperfusion after ET.

The median difference between the first occurrence of AWE and reperfusion was 0 days, so most patients showed AWE the first time when also reperfusion was visible for the first time [1. quartil = 4.7 months prior to the first showing of AWE/3. quartil = 24 months after the first showing of AWE].

8 of 37 patients (21.6%) needed a second endovascular treatment; three patients (8.1%) needed a third endovascular treatment.

In the group without AWE in any follow up after ET, 26 of 76 (34.2%) patients exhibited reperfusion. Three of 26 (11.5%) patients needed a second endovascular treatment, no patient needed a third endovascular treatment.

**Figure 2:**
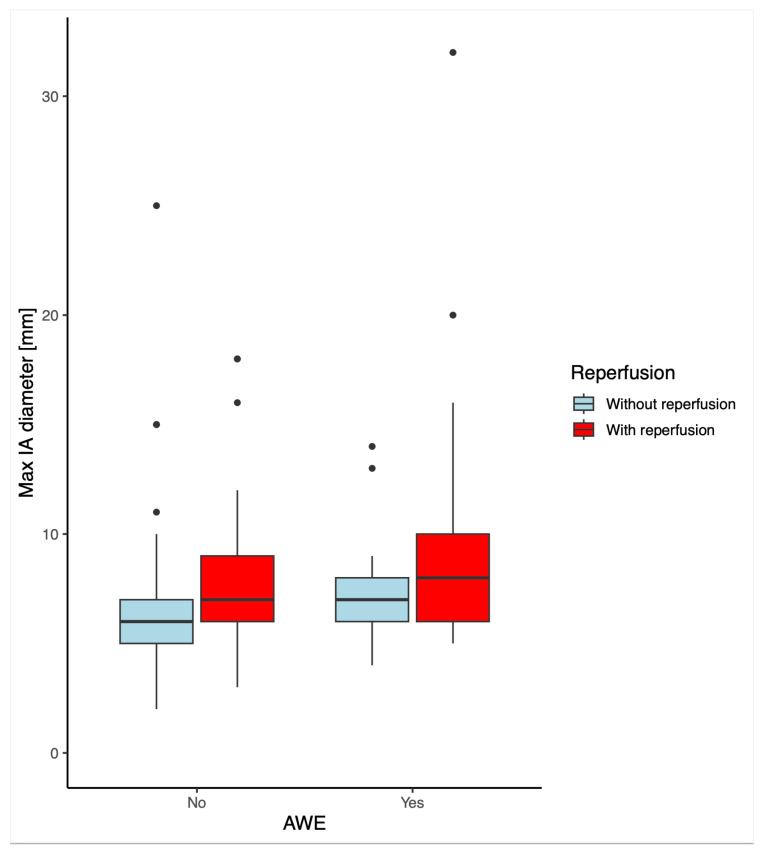
Boxplot of the maximum diameter of an IA vs. AWE in patients with and without reperfusion.

Demographics showed significant group-wise differences in age concerning AWE. Aneurysmal wall enhancement was statistically significantly different for patients with and without reperfusion (*p* < 0.001). The single maximum diameter was significantly higher for patients with reperfusion or AWE, this is visualized in the boxplots in Figure 2. The groups categorized for three grades of AWE showed statistically significant differences in all three AWE grade groups. However, there was no correlation between the AWE grade and reperfusion. There were also no statistically significant differences in PHASES scores for patients with or without AWE or reperfusion.

**Figure 3:**
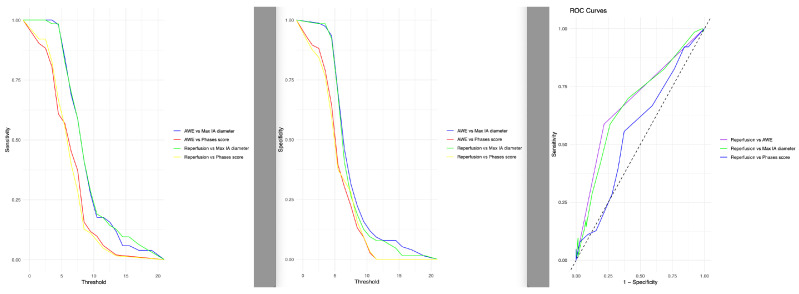
ROC–Curves showing the relation of the sensitivity and specificity of the maximum IA diameter, AWE, PHASES–Score and aneurysm reperfusion.

The ROC curve analysis results of the relations of sensitivity and specificity of the maximum IA diameter, AWE, PHASES score and reperfusion (numerical values in Table 5, visual display in Figure 3) indicates that AWE is not a complete biomarker for reperfusion with intermediate sensitivity (65.8%) and specificity (72.5%).

The logistic regression analysis reported that the maximum aneurysm diameter is associated with increased odds of reperfusion. For the maximum diameter as a predictor, the coefficient estimates with a *p*-value of 0.015, suggesting that it is significantly associated with reperfusion (*p* < 0.05). For AWE as a predictor, the coefficient estimates a *p*-value of <0.001, indicating a highly significant association with reperfusion. These results suggest that AWE has a stronger association with reperfusion than maximum aneurysm size.

No significant correlation was detected between the variables. There was no significant correlation between AWE and the grading of reperfusion.

## 4. Discussion

The understanding of the patophysiology of aneurysmal wall enhancement in IAs is crucial to identify unstable IAs and to plan further treatment options [23].

The results of this study show that increased AWE in non-ruptured IAs is common after ET, affecting 40% of patients and that increased AWE after ET is associated with reperfusion.

Although increased AWE in an untreated IA is widely regarded as a factor indicating aneurysm instability, there is still no clear consensus of its interpretation after ET, with some authors suggesting that for example in ET with stent–implantation, it might be due to flow diversion effects [18]. A recent study by Elsheikh et al. [17] proposed that increased AWE after endovascular treatment might reflect the healing process. Larsen et al. [24] showed that increased enhancement inside the aneurysmal cavity after ET is associated with stability and could be interpreted as a sign of aneurysm healing.

Although we cannot dispute these propositions and results, this study indicates that increased AWE after endovascular treatment is significantly associated with higher reperfusion rates after endovascular treatment in large aneurysms, so it might not be feasible to interpret it as a benign process. A recent study by Leber et al. [19] could also show that AWE after ET is associated with aneurysm recanalization/reperfusion, which is similar to our results. However, their patient cohort did also include patients who had initially suffered a subarachnoid hemorrhage in contrast to our study which is exclusively based on unruptured intracranial aneurysms.

The results of this study show that increased AWE could be considered as sign of persistent inflammation and consequent instability and at least trigger high alertness in assessing the adjacent angiography images.

The typical follow-up procedure for patients after endovascular treatment of IAs includes repeated MRI-scans to rule out and assess for possible complications after treatment and investigate for reperfusion or occurrence of new IAs. At our institution, patients usually receive several MRI-scans after ET, with usual intervals being in the initial first days after ET and subsequently after 6 and 12 months, followed by scans every 12 months. The decision to consider further DSA or retreatment is mainly based on the results of these scans.

If reperfusion is present, the rates of retreatment were comparable in patients with increased AWE and without increased AWE (28.6% in group 1 and 25% in group 2). This might be due to the fact that in the current evaluation and decision–making in interdisciplinary meetings, increased AWE is not considered and the indication for retreatment on MRI–angiography images and DSA studies.

However, in the subsequent statistical analysis, the data demonstrates that reperfusion after ET is only associated with AWE in large aneurysms.

This leads to the question: “What is a small and what is a large aneurysm?”. First of all we have to define which size parameters we include in our assessment. A lot of studies use the single maximum diameter; this is also the base and most important factor for calculationg the PHASES score [3]. In order to compare our results we also only assessed the single maximum diameter.

The exact threshold between large and small IAs is not yet clear and should be subject of further, preferably prospective studies. However, our data suggests that concerning the effects of AWE an initial size over 7.5 mm might be a sensible cut-off value, as based on our ROC analysis in Figure 3.

We also evaluated the AUC for different parameters, including reperfusion vs. the maximum aneurysm diameter (sensitivity of 71.6%, specificity of 60.0%), reperfusion vs. AWE (sensitivity of 73.4%, specificity of 58.7%, this further underlines that AWE after ET should trigger high alertness in assessing the subsequent angiography sequences (time of flight angiography or contrast MR angiography) but is not very specific as a single parameter.

Furthermore, these findings might suggest that the assessment of increased AWE is only feasible if the initially untreated IA is considered to be of a large caliber. Consequently follow–up examinations after ET should include VWI including contrast agents, which lengthens the imaging protocol but might ultimately contribute data in the difficult decision—making process of potential re-treatment after ET.

This finding is not entirely unexpected, since bigger IAs can be challenging to treat via ET and might therefore be more prone to aneurysm reperfusion.

In smaller aneurysms however, increased AWE does not seem to contribute information concerning possible reperfusion (as a marker for instability) and should be interpreted as an indeterminate parameter. Especially in these IAs, further research is highly warranted. This finding could lead to the conclusion that VWI after ET can be omitted in subsequent follow up scans, reducing scanning time and cost. Furthermore, a larger patient group could be examined which is not eligible for contrast agents (e.g., pregnancy or severe kidney dysfunction or allergies to MRI contrast agents).

However, aneurysms are in no way bound to a specific geometrical shape which leads to the conclusion that two aneurysms can have the same single maximum diameter but differ significantly in their shape and volume.

Unfortunately, it was not possible to retrieve 3 standardised dimensions for modeling the aneurysms as volumes in our data-set (which might be more accurate), as they were not always uniformly measured in more than the single biggest diameter in the DSA-studies of our cohort. However, we believe that adding a second or third dimension would also not entirely reflect the true specific geometrical shape of each aneurysm, given that they are often deformed and not uniformly shaped in standardised geometrical forms like cylinders or ellipsis.

To our knowledge, there is no internationally established grading system concerning AWE after treatment or prior to any treatment. In their study from 2020, Elsheikh et al. propose a grading system, ranging from faint to strong enhancement [17] but do not comment further on its clinical significance. We tried to further categorize AWE into 3 grades in this study, based on reproducible imaging characteristics. Although Grade 2 and 3 of AWE were significantly different concerning patients with and without aneurysm reperfusion, there was no correlation between the AWE grade and aneurysm reperfusion. This finding indicates that it is not only necessary to assess whether AWE is present but also its extent.

Since vessel walls of IAs are of a very small caliber, in our experience it can be difficult to assess AWE if faint and non linear (Grade 1 in our study) which is partly based on voxel size or suboptimal image quality. This might lead to diagnostic inaccuracy and inter-rater variability in reporting. However, the data of this study suggests that all grades of AWE were strongly associated with aneurysm reperfusion.

Although the PHASES score is very helpful concerning initial risk stratification of IAs, it does not predict which IAs are prone to reperfusion, although the biggest aneurysm diameter is an important contributing factor in calculating the score. This could lead to the conclusion that additional risk factors, which are reflected in the PHASES-Score (such as age, hypertension, site of aneurysm, population, history of SAH) are important criteria in initial assessment but lose their diagnostic value in decision making concerning re-treatment options, although it has to noted that only IAs that did not rupture were included in this study, which do not reflect the whole population of patients with IAs.

Our two main focus-groups concerning AWE showed significant differences in age, which we cannot fully explain. One possible contributing factor might be that athersclerosis (which can lead to increased AWE [12]) is more common in an older age-group. This should be further explored in a much larger patient cohort.

Limitations include the retrospective design, which includes no fixed time points for follow-up scans of each patient. Although usually a fixed schedule for follow-up scans is applied after ET (one day, 6 months, 12 months, 24 months, 48 months) the amount of scans can be increased based on the clinical course of each patient including newly arising symptoms or complications such as reperfusion or decreased if patients don’t comply with their fixed appointments. These facts cause heterogeneity of the intervals and number of follow-up scans after ET and also the time intervals from the initial treatment to the first MRI study including sequences for assessment of AWE. HR-VWI MRI sequences were introduced late in 2014 at our institution, with our analysis of HR-VWI starting with 1 January of 2015, but our database includes patients who received treatment earlier to introduction of these sequences. This leads to slight inaccuracies in the comparison of the first time AWE and reperfusion occured. Although these dates were fairly parrallel in most patients (median of 0 days difference between the first occurence of AWE and reperfusion) some patients already showed reperfusion prior to the introduction of HR-VWI in our institution. Moreover, in our clinical routine patients are not continually scanned on the same MRI-scanner, so the analyzed scans were performed on 2 different scanners (1.5 and 3 T), which also contributes to heterogeneity of image acquisition and image data. However, a study by Roa et al. [25] showed that there are no significant differences between different manufacturers or different field strengths (comparing 3 T and 7 T though, which differs from our study setting) concerning HR-VWI of IAs. 31 of 51 patients (60.8%) which showed AWE after treatment received their baseline and first post-treatment MRI-scan on the same MRI-scanner though, with no recognizable difference in enhancement characteristics. In conclusion, to our knowledge there is no difference in diagnostic sensitivity concerning HR-VWI in IAs due to field strength. Image quality of our MRI studies allowed evaluation of AWE in both sequence-types (T1 SPACE FS and T1 SE FS blood-suppression). The main limitation of T1-SPACE FS sequences is that they can be susceptible to motion artifacts due to the long acquisition time. The main limitation of T1 SE FS blood-suppression sequences is that they are only available in an axial plane. The two sequence types differ in their characteristics though, which also contributes to heterogeneity of the data.

Although our results clearly suggest that AWE is associated with reperfusion in large aneurysms, we believe that our results should be validated further in a prospective setting with fixed time points and on a single MRI-scanner.

Since only endovascularly treated IAs were included, there is a lack of correlation with histopathological data, which would have been helpful in interpreting increased AWE. This data might always be challenging to acquire though, since most patients with IAs receive endovascular treatment at our institution and also patients who do receive surgical clipping as a treatment option do not receive histopathological workup since the treated IAs are not resected.

Aneurysm reperfusion does also not always imply need for further endovascular retreatment—it would be interesting to launch a study with a longer period of observation (e.g., 10 years). In this current study the observation period was limited to up to 5 years. Furthermore, a study concerning reperfusion including patients who demonstrate increased AWE prior to any treatment and patients who do not might be interesting. Due to our small sample size of patients with increased AWE prior to any intervention, this could not be achieved in this study. Studies including radiomics–associated feature assessment of increased AWE in IAs could also be of great interest.

## 5. Conclusions

In contrast to other studies, we could show that commonly occurring increased AWE after ET of large IAs is associated with higher rates of aneurysm reperfusion, implying that AWE is a marker of instability after ET. However, in small IAs, increased AWE after ET is not associated with higher rates of aneurysm reperfusion.

Based on these results, in patients with large IAs it might be feasible to consider diagnostic re-angiography or early re-treatment if increased AWE is present after ET.

## Figures and Tables

**Table 1:** Inclusion and exclusion criteria.

Inclusion Criteria	Exclusion Criteria
Age above 18	Age below 18
One non-ruptured intracranial aneurysm	History of a ruptured intracranial aneurysm with subarachnoid hemorrhage
Two full MRI studies, if possible one study prior to endovascular treatment	Endovascular treatment via stent or stent—assisted coiling
Patient history including possible hypertension and ethnicity (for calculating PHASES—Score)	Patient history including vasculitis or intracranial vascular malformation

**Table 2:** Specific sequence parameters for HR–VWI. T = Tesla, TR = repetition time, TE = echo time, FA = flip angle, FOV = field of view, mm = millimeters, min = minutes. s = seconds, ms = milliseconds.

Sequence	T1 SPACE FS		T1 SE FS Blood-Suppression	
Field strength	1.5 T	3 T	1.5 T	3 T
Section orientation	Sagittal	Sagittal	Axial	Axial
TR; TE	550 ms; 7.2 ms	600 ms; 8.9 ms	641 ms; 16 ms	709 ms; 16 ms
FA	120	120	70	70
FOV	248 * 256	220 * 220	220 * 158	220 * 178
Matrix	224 * 256	440 * 440	512 * 368	512 * 416
Slice thickness	1 mm	1 mm	2 mm	2 mm
Scan time	5 min 8 s	6 min 10 s	2 min 5 s	3 min 3 s

**Table 3:** Population demographics and comparison of clinical and radiological characteristics between patients with and without reperfusion. * *p*-values using Fisher exact test. ^+^ *p*-value same as the Fisher exact test was computed between AWE and reperfusion.

	Reperfusion			
	Overall Sample (n = 127)	No (n = 64)	Yes (n = 63)	*p*-Value
Median age (IQR, 25–75)	59.0 [48.1 to 65.7]	58.7 [46.7 to 66.3]	59.5 [48.3 to 65.6]	*p* = 0.82
Female, n (%)	94 (74.0)	48 (75.0)	46 (73.0)	*p* = 1.0 *
Median PHASES score (IQR, 25–75)	5 [4 to 8]	5 [4 to 8]	6 [4 to 8]	*p* = 0.25
Median max IA diameter in mm (IQR, 25–75)	7.0 [6.0 to 9.0]	6.0 [5.0 to 8.0]	8.0 [6.3 to 10.0]	*p* < 0.001
AWE [Yes], n (%)	51 (40.2)	14 (27.5)	37 (72.5)	*p* < 0.001 *^+^
AWE [No], n (%)	76 (59.8)	50 (65.9)	26 (34.2)	*p* < 0.001 *^+^
AWE Grade 1, n (%)	17 (13.4)	6 (9.4)	11 (17.5)	*p* = 0.03 *
AWE Grade 2, n (%)	21 (16.5)	5 (7.8)	16 (25.4)	*p* = 0.001 *
AWE Grade 3, n (%)	13 (10.2)	3 (4.6)	10 (15.9)	*p* = 0.005 *

**Table 4:** Population demographic and comparison of clinical and radiological characteristics between patients with and without AWE. * *p*-values using Fisher exact test. ^+^ *p*-value same as the Fisher exact test was computed between AWE and reperfusion.

	AWE			
	Overall Sample (n = 127)	No (n = 76)	Yes (n = 51)	*p*-Value
Median age (IQR, 25–75)	58.9 [47.7 to 65.7]	55.7 [45.2 to 63.8]	61.2 [51.9 to 66.7]	*p* = 0.023
Female, n (%)	94 (74.0)	56 (73.7)	38 (74.5)	*p* = 1.0 *
Median PHASES score (IQR, 25–75)	5 [4 to 8]	5 [4 to 7]	6 [4 to 8]	*p* = 0.27
Median max IA diameter (IQR, 25–75)	7.0 [6.0 to 9.0]	6 [5.0 to 9.8]	8.0 [6.3 to 10.0]	*p* = 0.003
Reperfusion	63 [49.6]	26 [41.3]	37 [58.7]	*p* < 0.001 *^+^

**Table 5:** Receiver operating characteristic analysis of reperfusion and aneurysmal wall enhancement. Numbers within parentheses are 95% CI. AUC = area under the receiver operating characteristic curve, CI = confidence interval.

Method of Measurements	AUC	Cutoff	Youden’	Sensitivity (%)	Specificity (%)
Reperfusion vs. AWE	0.69	-	0.38	65.8	72.5
Reperfusion vs. max IA diameter	0.67	7.5 mm	0.33	73.4	58.7
Reperfusion vs. PHASES score	0.56	5.5	0.18	62.5	55.6
AWE vs. max IA diameter	0.64	7.5 mm	0.27	68.4	58.8

## Data Availability

The data will be made available upon a request.
